# Development of a temporally harmonized asset index: evidence from across 50 years of follow up of a birth cohort in Guatemala

**DOI:** 10.1186/s12874-021-01263-4

**Published:** 2021-04-26

**Authors:** Jithin Sam Varghese, John A. Maluccio, Solveig A. Cunningham, Manuel Ramirez-Zea, Aryeh D. Stein

**Affiliations:** 1 Nutrition and Health Sciences Program, Laney Graduate School, Emory University, Atlanta, GA USA; 2 Economics Department at Middlebury College, Middlebury College, Middlebury, VT USA; 3 Hubert Department of Global Health, Emory University, 1518 Clifton Rd NE, #7007, Atlanta, GA 30322 USA; 4 INCAP Research Centre for the Prevention of Chronic Diseases (CIIPEC), Institute of Nutrition of Central America and Panama (INCAP), Guatemala City, Guatemala

**Keywords:** Social mobility, Birth cohort, Life course epidemiology

## Abstract

**Background:**

Asset-based indices are widely-used proxy measures of wealth in low and middle-income countries (LMIC). The stability of these indices within households over time is not known.

**Methods:**

We develop a harmonized household asset index using Principal Component Analysis for the participants (*n* = 2392) of INCAP Longitudinal Study, Guatemala using data from six waves of follow-up over the period of 1965–2018. We estimate its cross-sectional association with parental schooling (in 1967–75) and attained schooling (in 2015–18) of cohort members. We study how patterns of cross-sectional loadings change over time and between urban-rural settings. We assess its robustness to omission of assets or study waves and alternate specifications of factor extraction procedure (exploratory factor analysis, multiple correspondence analysis).

**Results:**

The harmonized index constructed using 8 assets and 11 housing characteristics explained 32.4% of the variance. Most households increased in absolute wealth over time with median wealth (25th percentile, 75th percentile; households) increasing from − 3.74 (− 4.42, − 3.07; 547) in 1967 to 2.08 (1.41, 2.67; 1145) in 2017–18. Ownership of television, electricity, quality of flooring and sanitary installation explained the largest proportion of variance. The index is positively associated with measures of schooling (maternal: r = 0.16; paternal: r = 0.10; attained: r = 0.35, all *p* < 0.001). In 2015–18, house ownership versus housing characteristics and ownership of electronic goods differentiate households in urban and rural areas respectively. The index is robust for omission of assets or study waves, indicator categorization and factor extraction method.

**Conclusion:**

A temporally harmonized asset index constructed from consistently administered surveys in a cohort setting over time may allow study of associations of life-course social mobility with human capital outcomes in LMIC contexts. The approach permits exploration of trends in household wealth of the sample over a follow-up period against repeated cross-sectional surveys which permit the estimation of only the mean trajectory.

**Supplementary Information:**

The online version contains supplementary material available at 10.1186/s12874-021-01263-4.

## Key messages

- Stability of asset indices within households over time is not known.
- The constructed harmonized asset index demonstrates wealth gains over a 50-year period for most households in a birth cohort from a LMIC setting.
- The constructed harmonized asset index is robust for omission of assets or study waves, indicator categorization and factor extraction method.

## Background

Asset indices are widely used proxy measures of individual and household wealth in studies and surveys conducted in low and middle-income countries (LMIC) due in part to their ease of collection and estimation [1–3]. Such indices are proxy measures of ‘long-run’ income, poverty and wealth, and are closely associated with non-food expenditures [4, 5]. Patterns of asset ownership allow researchers to place households on a continuum along the wealth spectrum [6]. Validation studies show moderate to high associations of household asset indices with other manifest measures of socioeconomic status (such as schooling and income) and correlations with individual measures of health (such as childhood stunting and adult overweight) [4, 7–10]. Asset indices are expected to reveal wealth gains over time in growing economies. However, unavailability of longitudinal asset data over the life course is a significant barrier to exploring such gains for individuals or individual households [11]. The comparability of asset indices over time and between contexts is an active area of research in social sciences in the last decade [1, 3, 11–13].

The most commonly used procedure for development of an asset index for a sample consists of selection of items for inclusion, categorization of levels, specification of the correlation matrix and factor extraction [2]. A valid index developed should display theoretically-expected associations with external measures such as schooling, income or consumer expenditure. Potential problems in constructing an index include changing importance of assets over time reflected in item loadings for a pooled asset index versus cross-sectional indices, as well as the capacity of items to differentiate households (for example in what are often quite different urban versus rural settings). Given the paucity of longitudinal data in LMIC, as well as the scale and velocity of economic, demographic and epidemiological changes, it is important to understand patterns of wealth accumulation over the life course for 80% of the world’s population [14–16]. These patterns of wealth accumulation could reflect trajectories of social mobility which are associated with adult health outcomes.

In this paper, we develop and estimate an asset index, comprising durable assets and housing characteristics for members of a birth cohort (and their families) from four villages in rural Guatemala, harmonized over a 50-year period of study follow-up [17]. The Guatemalan economy is the largest in Central America, with modest growth rates of 3.5% per annum in the last five years. Guatemala is the fifth poorest country in the Latin America and Caribbean region, with high rates of poverty and inequality [18]. A low share of average tax burden, personal income taxes and social spending has led to inadequate provision of health, education and other public services. Since the onset of SARS-CoV2, Guatemala has experienced substantial loss of employment and disparities in access to schooling, worsening existing vulnerabilities [19, 20]. Alongside Guatemala as a whole, the study villages have undergone transformative social and economic changes in the last 50 years [21, 22]. Literacy and schooling outcomes have improved over the study period, on par with national averages. Road and transportation have also improved steadily over time resulting in better access to non-agricultural jobs. Over the duration of the study, GDP per capita (USD, 2010$) for Guatemala has risen from $1692 in 1967 to $3160 in 2018 [23]. We estimate its cross-sectional association with parental schooling and attained schooling of cohort members. We study how patterns of cross-sectional loadings change over time and between urban and peri-urban/rural settings at different points in time. We then assess the robustness of the benchmark index to omission of assets, study waves and to the use of alternative statistical methods.

## Methods

### Study population

The Institute of Nutrition of Central America and Panama (INCAP) conducted a cluster randomized trial in four rural villages matched on population size and density in the Department of El Progreso, Guatemala from 1969 to 1977 [24]. The INCAP Oriente Longitudinal Study in Guatemala is the longest running birth cohort in any LMIC [25, 26]. Two villages were randomly assigned to receive an energy and protein drink, Atole. The other two villages were assigned a low energy drink with all energy derived from sugar, Fresco. Details of the supplementation and study, as well as characteristics of the 2392 cohort members (comprising all individuals ages 0 to 7 living in the villages at any point during the 1969-77 study period) have been described previously [25]. The unit of observation in this study is the household in which a cohort member resided in each study wave.

### Data collection and variable specification

#### Durable assets and housing characteristics

Information on contextually appropriate durable assets which were contextually appropriate was collected from households with a cohort member residing in any of the four villages (as part of village censuses conducted) in the 1967, 1975, 1987 and 2002 study waves, and from households of all cohort members interviewed in the 2015–16 and 2017–18 study waves regardless of residential location (Supplementary Notes 1 and 2). Depending on the age and sex of the individual, it could be their own house, parents’ house or marital house. Cohort members were born between 1962 and 1977 so that in 1975 those who had been born were between 0 and 13 years old and in 2017–18 between 40 and 57 years old. Individual items were queried until they became irrelevant or negligible in value (e.g. record player) and additional new items including computer, telephone (fixed or cell phone) and washing machine added as they became available [6]. Only ownership of each item was collected and not information on the quantity, quality or functioning, technological generation and substitute assets.

Characteristics of the residence also were collected. These included ownership of house and land, number of rooms, material used for construction (floor, roof and wall), whether there was electricity, location of the kitchen, medium of cooking, sanitation, and sewage facilities. We categorized non-binary housing characteristics into low and high quality based on expert opinion. We created rooms per member, an indicator of crowding, such that a higher number reflects greater wealth [27]. We assume no information bias from self-report of ownership and housing characteristics [28]. Details are provided in Supplementary Table 1.

All participants gave written informed consent before participation. All methods were performed in accordance with the relevant guidelines and regulations.

#### Schooling

Attained years of schooling was collected for parents of cohort members. Attained schooling of cohort members was collected in adulthood during the 2015–16 and 2017–18 study waves. The participants were asked “What is the highest grade that you successfully completed?”

### Statistical analysis

#### Sample and changes in composition over survey waves

We compare early life characteristics (parental schooling, atole supplementation, year of birth and sex) of households of cohort members who resided in their original village in 1987 and 2002 versus households of cohort members who did not reside in the original villages. We also compared those interviewed and not interviewed in recent waves (2015–16, 2017–18). We do not have information on the households of cohort members in 1987 or 2002 if they were not residing in their original study village at the time.

#### Construction of the harmonized asset index

For greater comparability to previously published work, we included ownership (yes/no) of radio, record player, sewing machine, refrigerator, television, bicycle, motorcycle and automobile. We included house ownership, land ownership, rooms per member, quality of housing construction (floor, roof, walls), whether the house had a separate kitchen, formal cooking medium, sanitary installation, improved water source and availability of electricity [17, 29]. We imputed ownership of land, record player, sewing machine, television, motorcycle and automobile as zero for the 1967 wave when they were not asked. We imputed ownership of record player for 2002 and onwards as zero. We pooled all study waves (1967, 1975, 1987, 2002, 2015–16, 2017–18) into a single dataset for the main analyses. Since siblings were included in the original cohort in early waves, the number of households does not equal number of cohort members. The 2392 individuals recruited during the period 1969–77 come from 816 unique households. In the 2015–16 and 2017–18 waves, 176 and 240 individuals from the 1163 and 1265 who were followed up are married to each other. We therefore include household as the unit of observation.

Various approaches for constructing asset indices have been described in the literature, of which the most common is principal component analysis (PCA) [5]. PCA is a statistical procedure that projects data points from the real number space onto a set of orthogonal ‘principal components’ such that the first component explains the maximum variance in the original data, and each subsequent component explains the maximum remaining variance. We performed PCA on a correlation matrix created from the pooled dataset of binary variables comprising ownership of durable assets, housing characteristics and crowding as a continuous variable. We retained the first component from the PCA as the harmonized asset index [2, 17]. Some research has explored the potential of higher order principal components to explain other dimensions of wealth (such as agricultural wealth). Because these components are uncorrelated with the first principal component and in this context did not display interpretable loadings for housing characteristics, we did not consider them [5, 30].

We visually assessed the empirical distributions for clumping and truncation, examining histograms for each study wave [3]. Clumping occurs when many households have the same value of index due to limited variation in ownership and housing characteristics. Truncation is the failure to differentiate between relatively low or high levels. Both of these phenomena are ideally resolved by including additional suitable assets or characteristics (or quality, quantity or other information about them) which could differentiate at points along the distribution of the index.

Usage of PCA with binary variables has been criticized for violating assumptions of linearity and normality. Although PCA does not impose constraints on each variable, it assumes a multivariate normal distribution of the variables for components to be independent. Alternative procedures have their corresponding strengths and limitations. For instance, Multiple Correspondence Analysis (MCA), which is a suitable alternative to PCA for categorical data cannot be used with continuous data. Polychoric/tetrachoric PCA assumes bivariate normal distributions between latent variables which form the observed discrete variables. In practice, these methods tend to produce indices that are highly correlated [31]. We assessed correlation of the harmonized asset index with cross-sectional schooling-related measures of SES among cohort members (parental schooling in 1967–75 and own attained schooling in 2015–16 or 2017–18).

#### Sensitivity analysis

We also constructed cross-sectional indices (S1) with the same set of indicators used in the harmonized index, stratifying by region of residence (urban, rural) of cohort members in the final two waves. We also assessed the Spearman rank correlation of the harmonized index with a separate index constructed by including newer assets introduced in 2002 and later (S2; video player, sound system, computer, telephone, washing machine and sewage system) after imputing the newer assets as zero for earlier waves (1987 and before).

We report the Spearman rank correlations of the harmonized index with alternative indices to assess the sensitivity to dropping assets and study waves (S3), the structure (S4) of the correlation matrix (Pearson, polychoric) and the factor extraction method (PCA, Exploratory factor analysis, MCA), categorization of housing characteristics into ordinal (S5; low, medium, high). Exploratory factor analysis assumes an underlying factor which give rise to the observed distribution of assets and housing characteristics. MCA is a generalization of PCA when variables are categorical. We converted crowding into a binary variable for the MCA with values greater than 0.75 rooms per person set to 1 and otherwise 0. Additional information on the various sensitivity analyses is provided in Supplementary Note 3. We carried out our analysis using R 3.5.1 and tidyverse 1.3.0 [32, 33].

## Results

### Comparison of baseline characteristics of cohort members

Data on assets were available for 547 households of cohort members in 1967 and 755 in 1975 (totalling 1302), covering 2073 of the 2392 cohort members. Durable asset and housing information was unavailable for the remaining 319 individuals in both 1967 or 1975. Information on cohort members by survival and participation in 1987 and later study waves is available in Table 1. Of the original members, 2023 were known to be alive, and data on 1388 residing in Guatemala were collected in the 2015–16 or 2017–18 waves. The majority of the deceased died prior to 1987 (236 out of 385), most in early childhood. Households of cohort members who died were similar in wealth to those who were alive (as measured by the harmonized index at baseline, described below). In 1987, compared to those for whom asset data is available, those for whom data is unavailable were older (born in 1968 vs 1972) and more likely to be female (56.8% vs 44.2%). Also compared to those for whom asset data is available, those for whom asset data is unavailable in 2002, 2015–16 and 2017–18 had fathers with higher median attained schooling (2 vs 1 years) compared to those whose asset data is available. Cohort members with and without asset data were otherwise similar to those whose data is unavailable (study wave; 2002, 2015–16, 2017–18).

**Table 1:** Characteristics of cohort members (*n* = 2392) by life status and availability of asset data^a^ at different waves of study

	Alive and asset data available				Alive and asset data unavailable				Died prior to study wave			
Early life characteristics	1987 (***n*** = 1360)	2002 (***n*** = 1053)	2015–16 (***n*** = 1163)	2017–18 (***n*** = 1265)	1987 (***n*** = 796)	2002 (***n*** = 1037)	2015–16 (***n*** = 860)	2017–18 (***n*** = 739)	1987 (***n*** = 236)	2002 (***n*** = 302)	2015–16 (***n*** = 369)	2017–18 (***n*** = 385)
Paternal Schooling (y)^b^	1 [0;3]	1 [0;3]	1 [0;3]	1 [0;3]	0 [0;3]	2 [0;3]	2 [0;3]	2 [0;3]	1 [0;2]	0 [0;2]	0 [0;2]	0 [0;2]
Maternal Schooling (y)^c^	1 [0;2]	0 [0;2]	0 [0;2]	1 [0;2]	0 [0;2]	1 [0;2]	1 [0;2]	1 [0;2]	0 [0;2]	0 [0;2]	0 [0;2]	0 [0;2]
Household wealth in 1967-75 (z-scores)^d^	−3.6 ± 1.1	−3.7 ± 1.1	−3.6 ± 1.0	−3.7 ± 1.0	−3.7 ± 0.9	−3.5 ± 1.0	−3.6 ± 1.0	−3.5 ± 1.0	−3.6 ± 1.1	−3.6 ± 1.0	−3.6 ± 1.0	−3.6 ± 1.0
Atole supplemented	52.0%	50.5%	54.3%	52.7%	54.3%	54.5%	50.1%	52.2%	55.1%	57.0%	55.8%	55.6%
Year of Birth (19XX)	72 [68;74]	71 [67;74]	70 [67;74]	70 [67;74]	68 [66;71]	70 [67;73]	70 [67;74]	71 [67;74]	73 [70;75]	72 [69;75]	72 [68;74]	71 [68;74]
Female	44.2%	46.8%	60.1%	55.6%	56.8%	52.5%	36.9%	40.1%	46.2%	41.4%	39.6%	41.6%
Attained Schooling (y)^e^			5 [2;6]	6 [2;6]			6 [2;6]	6 [2;6]				2 [0;4]

^a^Asset data can be missing due to one of the following: non-response (all waves), migrated outside study villages in Department of El Progreso (1987, 2002), migrated outside Guatemala (all waves); ^b^Available for 2041 cohort members; ^c^Available for 2169 cohort members; ^d^ from harmonized wealth index closest to birth; e Attained schooling for alive and not included in study wave from latest wave of availability (N; 2015–16: 232, 2017–18: 122) and for those dead between 2015 and 16 and 2017–18 (*N* = 15); Continuous variables are displayed as mean ± standard deviation (if normally distributed) or median [25th percentile, 75th percentile]. Categorical variables are displayed as percentage (%). Number of households with cohort members who were alive and for whom asset data were available: 617 (in 1987), 820 (in 2002), 1075 (in 2015-16) and 1145 (in 2017-18)

### Durable assets and housing characteristics

Living standards in households of cohort members improved over time (Table 2). Ownership of electronic goods such as a television or refrigerator increased over time, especially from 1987 onward, while the proportion owning a radio increased until 1987 and then later decreased. Ownership of motor vehicles (motorcycle, automobile) also increased over time. Ownership of the house has been consistently high (84% in 1967 and 83% in 2017–18). However, the average number of rooms per member nearly doubled from 0.61 in 1967 to 1.13 in 2017–18. Land ownership decreased from 1975 (74%) to 2002 (53%) and subsequently increased (67%). We observed a large increase in sanitation (5% in 1967 to 99% in 2017–18). Only a small proportion of households (11%) had electricity in 1975 but nearly all (97%) did by 2017–18. Ownership of appliances like refrigerator (1% in 1967 to 71% in 2017–18), television (1% in 1975 to 92% in 2017–18) and computer (1% in 2002 to 31% in 2017–18) also increased over time. By 2017–18, nearly all households (96%) possessed a telephone (fixed or cell phone).

**Table 2:** Ownership of assets and housing characteristics across cohort households surveyed as part of INCAP Oriente Longitudinal Study (1967–2018) by study wave

	1967 (***n*** = 547)	1975 (***n*** = 755)	1987 (***n*** = 617)	2002 (***n*** = 820)	2016 (***n*** = 1075)	2018 (***n*** = 1145)
Radio	0.32	0.53	0.58	0.21	0.30	0.22
Record Player	n.a.	0.02	0.06	n.a.	n.a.	n.a.
Sewing Machine	n.a.	0.11	0.10	0.10	0.19	0.18
Refrigerator	0.01	0.02	0.04	0.27	0.67	0.71
Television	n.a.	0.01	0.22	0.77	0.92	0.92
Bicycle	0.01	0.03	0.11	0.53	0.51	0.45
Motorcycle	n.a.	0.00	0.01	0.01	0.24	0.31
Automobile	n.a.	0.00	0.01	0.08	0.27	0.27
Video player	n.a.	n.a.	n.a.	0.09	0.55	0.47
Sound system	n.a.	n.a.	n.a.	0.38	0.59	0.57
Computer	n.a.	n.a.	n.a.	0.01	0.34	0.30
Telephone	n.a.	n.a.	n.a.	0.31	0.95	0.96
Washing machine	n.a.	n.a.	n.a.	n.a.	0.19	0.21
Owns land	n.a	0.74	0.80	0.53	0.73	0.67
Owns house	0.84	0.80	0.82	0.78	0.81	0.83
Rooms per member	0.61	0.61	0.60	0.77	1.09	1.13
High quality floor	0.04	0.11	0.37	0.76	0.90	0.91
High quality roof	0.73	0.72	0.89	0.98	0.99	0.99
High quality walls	0.46	0.56	0.81	0.93	0.98	0.98
Separate kitchen	0.58	0.73	0.90	0.91	0.95	0.97
Formal cooking medium	0.01	0.46	0.67	0.83	0.91	0.87
Sanitary installation	0.05	0.16	0.57	0.89	0.98	0.99
Electricity	0.00	0.11	0.72	0.96	0.98	0.97
Improved water source	0.03	0.10	0.42	1.00	0.98	0.97
Improved sewage system	n.a.	n.a.	n.a.	0.11	0.52	0.54

The first four waves of data collection include households of cohort members residing in original study villages (1967, 1975, 1987, 2002) and the last two waves are surveys of households of cohort members living anywhere in Guatemala (2015–16, 2017–18). Details of the categorization schema for assets and housing characteristics are provided in Supplementary Table 1

### Harmonized index construction

Table 3 shows loadings on each indicator for the harmonized index in the first column and, then indices constructed for sensitivity analyses. The first principal component of the harmonized index explained 32.4% of the variance in the pooled data. Standardized loadings of the principal components are positive for ownership of each individual asset except radio (− 0.05). The largest positive loadings were for television (0.34), high quality floor (0.33), sanitary installation (0.33) and electricity (0.33). The lowest loadings were for record player (0.004), house ownership (0.03) and land ownership (0.07). Cronbach’s alpha for the items and Kaiser-Meyer-Olkin (KMO) measure of sampling adequacy were 0.84 and 0.91 respectively.

**Table 3:** Loadings on first principal component of the harmonized index, within each study wave, and stratified by urban or rural residence in middle adulthood

	Harmonized	Cross-sectional						Rural		Urban	
		1967	1975	1987	2002	2015–16	2017–18	2015–16	2017–18	2015–16	2017–18
Radio	−0.05	0.25	0.25	0.25	0.07	0.06	0.03	0.04	0.02	0.11	0.08
Record Player	0.00^a^	–	0.18	0.26	–	–	–	–	–	–	–
Sewing Machine	0.12	–	0.26	0.21	0.21	0.22	0.19	0.20	0.20	0.21	0.18
Refrigerator	0.28	0.24	0.28	0.20	0.35	0.40	0.37	0.39	0.35	0.36	0.39
Television	0.34	–	0.17	0.37	0.36	0.34	0.37	0.35	0.37	0.21	0.31
Bicycle	0.21	0.18	0.17	0.22	0.21	0.11	0.04	0.06	0.03	0.24	0.09
Motorcycle	0.17	–	0.19	0.09	0.09	0.25	0.22	0.26	0.24	0.18	0.15
Automobile	0.18	–	0.14	0.11	0.22	0.31	0.26	0.27	0.24	0.32	0.30
Owns land	0.07	–	0.05	0.18	0.19	0.21	0.17	0.15	0.15	0.36	0.29
Owns house	0.03	0.02	0.07	0.18	0.24	0.21	0.17	0.19	0.20	0.37	0.26
Rooms per member	0.17	0.24	0.21	0.08	0.18	0.22	0.20	0.21	0.18	0.25	0.24
High quality floor	0.33	0.33	0.33	0.32	0.31	0.33	0.38	0.36	0.38	0.21	0.31
High quality roof	0.20	0.32	0.28	0.24	0.15	0.10	0.15	0.13	0.19	0.04	−0.02
High quality walls	0.26	0.42	0.35	0.29	0.27	0.12	0.27	0.23	0.27	−0.02	0.25
Separate kitchen	0.20	0.38	0.33	0.25	0.12	0.18	0.11	0.15	0.08	0.27	0.23
Formal cooking medium	0.28	0.34	0.31	0.24	0.30	0.28	0.30	0.28	0.28	0.21	0.34
Sanitary installation	0.33	0.35	0.26	0.25	0.24	0.21	0.21	0.2	0.21	0.17	0.17
Electricity	0.33	0.04	0.10	0.28	0.34	0.26	0.25	0.28	0.27	0.17	0.05
Improved water source	0.32	0.13	0.05	0.07	–	0.13	0.17	0.12	0.18	0.16	0.14
**% Variance explained by PC1**	**32.4**	**19.5**	**17.5**	**18.3**	**15.9**	**15.2**	**16.4**	**15.4**	**17.0**	**16.6**	**15.9**
**Households (n)**	**4959**	**547**	**755**	**617**	**820**	**1075**	**1145**	**773**	**816**	**302**	**329**

^a^ Loading for Record Player in Harmonized index is 0.004

**Fig. 1:**
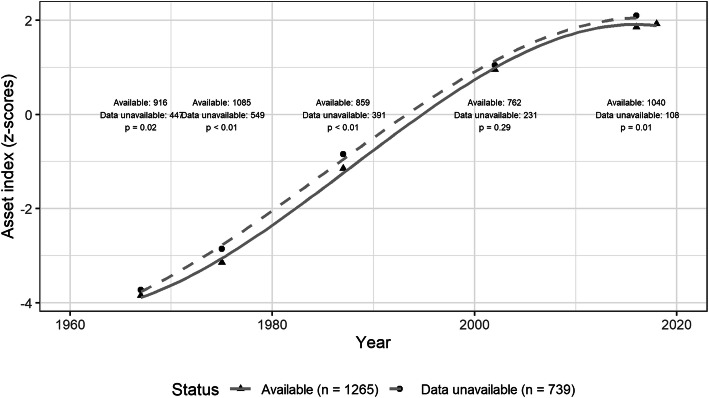
Absolute asset index over time of cohort members who are alive by data availability in 2017–18 (*n* = 2004). Asset index data is available for 1967, 1975, 1987, 2002, 2015–16 and 2017–18 with non-monotone missingness. Cubic splines represent mean population trajectory for those who participated and did not participate in study waves (fit using ggplot2 3.3.0). *P*-values displayed are from t-tests at each study wave

Visual inspection of histograms (Supplementary Figure 1) of the benchmark harmonized index (pooled, and separately by study wave) indicates clumping (limited range of data values) for early study waves (1967, 1975 and 1987). The index displayed truncation at the lower tails of the distribution in the early study waves but not for later ones or for the upper tails of the distributions. A summary of harmonized asset index scores across study waves is presented in Table 4. As households acquired additional assets the mean increases across study waves. The mean harmonized index score in the observed sample increased from − 3.76 in 1967 to 1.92 in 2017–18 while the SD increased from 0.91 in 1967 to 1.51 in 1987 but then declining to 1.09 by 2017–18. The inter-quartile range (3rd quartile – 1st quartile) of harmonized index scores increased from 1.35 in 1967 to 2.01 in 1987, after which it decreased to 1.26 in 2017–18. Most cohort members (99.8%) observed in 2017–18 experienced gains in the absolute level of the harmonized index for their household in adulthood relative to early life (Fig. 1). For those who did not participate in 2017–18, we observe a similar increase to the wave in which they were last observed. The harmonized index is positively associated with measures of schooling, another important indicator of socio-economic status: maternal schooling (with 1967 or 1975; r = 0.16), paternal schooling (with 1967 or 1975; r = 0.10) and attained schooling in adulthood (with 2015–16 or 2017–18; r = 0.35).

**Table 4:** Summary of harmonized index by study wave

Study wave	N	Mean ± SD	Median (IQR)	Range [Min, Max]
1967	547	−3.76 ± 0.91	−3.74 (−4.42, −3.07)	[−5.24, 0.23]
1975	755	−2.95 ± 1.28	−2.94 (−4.07, −2.25)	[−5.25, 2.45]
1987	617	−1.11 ± 1.51	−0.99 (−2.19, −0.18)	[−5.17, 2.8]
2002	820	0.95 ± 1.15	1.00 (0.33, 1.67)	[−3.84, 3.55]
2015–16	1075	1.86 ± 1.08	2.02 (1.27, 2.64)	[− 3.25, 4.15]
2017–18	1145	1.92 ± 1.09	2.08 (1.41, 2.67)	[− 3.44, 4.37]
Harmonized	4959	0.00 ± 2.48	0.78 (− 2.29, 2.08)	[− 5.25, 4.37]

### Sensitivity analysis

Cross-sectional indices constructed separately for each individual study wave using the same set of assets (S1; r ≥ 0.91) and urban-rural stratified indices for each of the final two waves (r ≥ 0.90) were also correlated with the harmonized index. We display the loadings of these different cross-sectional indices in Table 3 and their correlations with benchmark harmonized index in Table 5. An index including newer available assets (S2; video player, sound system, computer, telephone, washing machine and sewage) was correlated (r ≥ 0.91) with the benchmark harmonized index.

**Table 5:** Sensitivity analysis assessing associations of the harmonized index with alternate methodologies for creating the index, INCAP Oriente Longitudinal Study 1967–2018

**Correlation with cross-sectional asset index (S1)**		
**Study wave**	**Households**	**Rank correlation**
1967	547	0.95
1975	755	0.95
1987	617	0.91
2002	820	0.97
2015–16	1075	0.97
2017–18	1145	0.96
2015–16	773	0.97
Urban 2015–16	302	0.90
Rural 2017–18	816	0.95
Urban 2017–18	329	0.92
**Addition of newer assets to harmonized index**^**a**^ **(S2)**		
2002	820	0.91
2015–16	1075	0.91
2017–18	1145	0.92
**Alternative specification of correlation matrix and factor extraction method (S4)**		
PCA with Polychoric correlation	4959	0.96
Exploratory Factor Analysis^b^ with Pearson correlation	4959	1.00
Exploratory Factor Analysis^b^ with Polychoric correlation	4959	0.96
Multiple Correspondence Analysis^c^	4959	1.00
**Alternative categorization of housing characteristics into high, medium and low (S5)**		
PCA with Polychoric correlation	4959	0.96
Exploratory Factor Analysis^b^ with Polychoric correlation	4959	0.92

^a^ Video player, sound system, computer, telephone, washing machine, and improved sewage system were included and imputed as 0 for those waves (1987 and before) during which it was not collected; ^b^ EFA with varimax rotation and 1 factor extracted using residual minimization; ^c^ MCA was calculated with crowding > 0.75 set to 1 and otherwise 0; All rank correlations are significant (*p* < 0.001). Details of the categorization schema for assets and housing characteristics are provided in Supplementary Table 1. Polychoric correlation was calculated using psych package (v1.8.10)

The harmonized index was robust to omission (S3) of any pair of assets (r ≥ 0.97; Supplementary Table 2), any one or two study waves (r ≥ 0.96; except for omission of 1967 and 1975 where r = 0.91; Supplementary Table 3) and joint omissions of each single asset with each study wave (r ≥ 0.95; Supplementary Table 4). These results suggest that the index is stable even when we do not include assets or study waves such that an index created from a sparser dataset would be largely similar to the benchmark index. Alternative specifications of the correlation matrix and factor extraction methods (S4) on the pooled sample indicated a high correlation (Table 5) with the harmonized index based on PCA (range: 0.96–1.00). Asset indices constructed by re-specifying housing characteristics (as described in Supplementary Table 1) into three categories (S5; low/medium/high) were also highly correlated (PCA: 0.96, EFA: 0.92) with the original index.

Comparing cross-sectional indices for survey waves when cohort members were in adulthood (2002, 2015–16, 2017–18), ownership of refrigerator (loadings: 0.35 to 0.40), television (loadings: 0.34 to 0.37) and high quality flooring (loadings: 0.31 to 0.38) have the highest loadings. In general from 1967 to 2017–18, the loadings of housing characteristics such as the roof and walls decrease while those of assets increase. However, items such as house ownership (2015–16; loading = 0.37) and land ownership (loading = 0.36) have high loadings for the urban sample. Ownership of a television (loading = 0.35) and high quality floor (loading = 0.36) have high loadings within rural sample.

## Discussion

We attempted to develop a temporally harmonized asset index from consistently administered surveys in a cohort setting. Such an index can be used to study the impact of socio-economic mobility on measures of human capital in adulthood. For cohort members followed over a period of 50 years, an asset index created by pooling study waves shows an increase in absolute wealth over time. The constructed harmonized asset index was robust to various sensitivity analyses. Our analysis demonstrates wealth gains over time in a birth cohort from a LMIC setting. A harmonized index for a birth cohort is an improvement over repeated cross-sectional surveys because it permits the estimation of both the population mean trajectory, quantifying cross-sectional variation within the cohort and understanding trends in household wealth of the analytic sample over the follow-up period. Additionally, a harmonized index allows examination of trajectories of absolute wealth mobility over the life course and sensitivity of timing of wealth gains for human capital [34].

The results suggest a divergence or increased inequality in household wealth from 1967 to 1987 followed by a partial convergence in 2002. The observed pattern could reflect the transition of cohort members residing in the villages (typically in their parental homes) until adolescence and then the process of forming their own households. The period from 1987 to 2002 was marked with economic changes such as the transition from agricultural to non-agricultural jobs, increased access to electricity, piped water and increased ownership of electronic appliances such as televisions [22].

A cross-sectional analysis of item loadings demonstrate how importance of housing characteristics and assets in differentiating households changes over time. The temporally harmonized index was highly correlated with cross-sectional indices for each study wave on its own. Cross-sectional comparisons of urban and rural households indicate how house ownership differentiates households in urban areas while housing characteristics and ownership of electronic goods are better differentiators in rural areas. This potentially reflects the higher cost of owning houses in urban areas rendering it a stronger indicator of wealth. Similar to a study from Zimbabwe, we observed correlations of the pooled index with indices stratified by rural (and urban) residence [35]. Despite the few observed differences, the loadings are similar in magnitude over time and between settings for most items included such that developing a temporally harmonized index was feasible in our sample.

The index displayed internal consistency (or monotonicity) such that loadings for all assets, except radio, were positive [11]. The largest loadings were for electricity, television, high quality flooring and sanitary installation. Descriptive analysis shows that ownership of radios increased until 1987 and subsequently decreased reflecting changing consumption patterns. The index was robust to dropping items, study waves, alternate indicator categorization or specification of the correlation matrix and factor extraction method. The stability of the index despite dropping pairs of items indicates that at least in our sample, exclusion of infrastructure items (such as electricity) or housing characteristics do not change our results. The lower correlation when excluding 1967 and 1975 (r = 0.91) is likely due to fewer households having televisions, separate kitchens and high quality roofing in those waves – resulting in different loadings before and after exclusion. Consistent with earlier studies from other settings, indices derived from alternative procedures including EFA, MCA or polychoric PCA are highly correlated with the PCA-derived index suggesting that the final selection of which method to use does not matter substantively [2, 16, 31, 36]. The index displayed construct validity with external measures as shown by positively associations with both parental and attained schooling, consistent with previous results from Latin America [37]. Overall, the results from sensitivity analyses are consistent with previous research on stability of such indices in similar contexts [3, 11, 31, 38–40].

Our index has limitations inherent to the nature of data and methodology. A limited set of assets is available, computations need to be repeated on addition of future rounds, characteristics of assets are not available (quality or functioning, quantity, technological generation, substitute assets) and potential conflation of within year and between year variance [30]. The index displayed clumping and truncation at lower values in the early waves. The four study villages in 1967 and 1975 were similar to other poor rural areas of Guatemala at the time. Solving issues of clumping and truncation, however, would require addition of assets that increase variability at points along the index. Unfortunately, we did not have such assets which were collected during these study waves. Expanded assets, however, do not change rank order in a country which has experienced economic change similar to other countries. Measures of rural wealth such as farmland or pastoral land, agricultural equipment and livestock which could differently represent both pooled and cross-sectional rankings were not available [11, 41]. Previous research conducted towards developing a multidimensional poverty index suggested that consumer durables are able to sufficiently differentiate households in rural areas [42]. Barring these limitations, our index provides a descriptive understanding of trends in wealth in a cohort studied consistently for over 50 years from a growing economy.

## Conclusions

Our approach enables examination of the association of absolute (material pathways) and relative (psychosocial pathways) wealth mobility over the life course with other important outcomes including health and well-being [43, 44]. There are increasing numbers of longitudinal studies in LMIC settings and our study provides guidance for researchers for assessing long-term trends in household wealth. Our research suggests that consistently-administered asset indices are useful to study associations of changes in wealth in relation to human and social capital development over time in a cohort setting. We encourage researchers working in LMICs to collect contextually relevant, consistent measures of wealth.

## Supplementary Information

**Additional file 1.**

## Data Availability

The datasets generated and/or analysed during the current study are not publicly available. There are ethical or legal restrictions on sharing a de-identified data set. We cannot anonymize the data from this cohort as all individuals come from one of four previously named villages and hence are readily re-identifiable once their demographic characteristics are known. We will not post data to a public archive, but we will make a replication data set available to bona fide researchers who agree to sign an LDUA and are covered under an IRB. Please contact the Research Center for the Prevention of Chronic Diseases (CIIPEC) at the Institute of Nutrition of Central America and Panama for requests. The local data protection manager is Dina Roche (email: droche@incap.int; phone: + 502 5499 7220). The code is available at https://github.com/jvargh7/incap-asset-index.

## Abbreviations

DHS: Demographic and Health Survey
INCAP: Institute of Nutrition of Central America and Panama
LMIC: Low and Middle Income Country
PCA: Principal Component Analysis
SES: Socio-economic status
