# Supplementary material for: Development of a temporally harmonized asset index: evidence from across 50 years of follow up of a birth cohort in Guatemala
Source: BMC Med Res Methodol. 2021 Apr 26;21:85. doi: 10.1186/s12874-021-01263-4 (PMC8074514; doi:10.1186/s12874-021-01263-4)
Supplement: Supplementary file 1 — Additional file 1. [file 12874_2021_1263_MOESM1_ESM.docx]

**Supplementary Note 1: Questionnaire on ownership of durable assets and housing characteristics in 2017-18 and corresponding availability in prior study waves**

The next set of questions is about where you have lived in the past 3 months.

Note: If the cohort member lives in a university or employment residence, the following questions only refer to their room or apartment in the residence.

| Question | Options | 1967 | 1975 | 1987 | 2002 | 2015-16 | 2017-18 |
| --- | --- | --- | --- | --- | --- | --- | --- |
| Number of persons in the house |  | 🗸 | 🗸 | 🗸 | 🗸 | 🗸 | 🗸 |
| Ownership of land | No  Yes  Do not know  Not responded  Not applicable |  | 🗸 | 🗸 | 🗸 | 🗸 | 🗸 |
| Legal title of house | No  Yes  Do not know  Not responded  Not applicable | 🗸 | 🗸 | 🗸 | 🗸 | 🗸 | 🗸 |
| Number of rooms |  | 🗸 | 🗸 | 🗸 | 🗸 | 🗸 | 🗸 |
| Floor | 1, Earth  2, Brick or clay  3, Cement cake  4, Mosaic  5, Wood  Do not know  Not responded  Not applicable | 🗸 | 🗸 | 🗸 | 🗸 | 🗸 | 🗸 |
| Roof | 1, Thatched or similar material  2, Tile  3, Metal  4, Concrete  6, Tile brand - Duralite | 🗸 | 🗸 | 🗸 | 🗸 | 🗸 | 🗸 |
| Wall | 1, Cane or similar material  2, Mix of clay, cane and wood  3, Mix of clay, cane wood and cement  4, Mud brick  5, Mud brick with cement  6, Wooden  7, Brick  8, Metal | 🗸 | 🗸 | 🗸 | 🗸 | 🗸 | 🗸 |
| Location of kitchen | 0, No kitchen  1, In the bedroom  2, In separate place  3, Built-in housing | 🗸 | 🗸 | 🗸 | 🗸 | 🗸 | 🗸 |
| Method of cooking | 0, Without means to cook  1, On the floor  2, Low removable wood and charcoal stove  3, High removable wood and charcoal stove  4, Low fixed wood and charcoal stove  5, High fixed wood and charcoal stove  6, Wood stove  7, Gas stove  8, Electric stove | 🗸 | 🗸 | 🗸 | 🗸 | 🗸 | 🗸 |
| Electricity | No  Yes  Do not know  Not responded  Not applicable | 🗸 | 🗸 | 🗸 | 🗸 | 🗸 | 🗸 |
| Sanitary installation | 1, No sanitary installation  2, Toilet  3, Latrine  4, Septic tank  5, Toilet  6, Other  7, Pit latrine | 🗸 | 🗸 | 🗸 | 🗸 | 🗸 | 🗸 |
| Sewage system | 1, No sewage drain  2, Cesspit system  3, Sewerage system  4, Septic tank  5, Piping for a stream | 🗸 | 🗸 | 🗸 | 🗸 | 🗸 | 🗸 |
| Water source | 1, Spring or river  2, Public pile or full of pitchers  3, Well in the house or neighborhood  4, Public water system in the house | 🗸 | 🗸 | 🗸 | 🗸 | 🗸 | 🗸 |
| Garbage disposal | 1, Throw in yard  2, Bury it  3, Burn  4, Throw into ravine  5, Public dump  6, Other |  |  |  |  | 🗸 | 🗸 |

THE PROPERTY OF THE HOUSE

Now we are going to talk about the characteristics and assets of the house where you live.

| Question | Options | 1967 | 1975 | 1987 | 2002 | 2015-16 | 2017-18 |
| --- | --- | --- | --- | --- | --- | --- | --- |
| Telephone/Fixed line | No  Yes |  |  |  | 🗸 | 🗸 | 🗸 |
| Cell phone | No  Yes |  |  |  | 🗸 | 🗸 | 🗸 |
| Radio | No  Yes | 🗸 | 🗸 | 🗸 | 🗸 | 🗸 | 🗸 |
| Sound equipment/CD player | No  Yes |  | 🗸 | 🗸 | 🗸 | 🗸 | 🗸 |
| Ipod /Ipad | No  Yes |  |  |  |  |  | 🗸 |
| TV | No  Yes |  | 🗸 | 🗸 | 🗸 | 🗸 | 🗸 |
| Video / DVD player | No  Yes |  |  |  | 🗸 | 🗸 | 🗸 |
| Computer | No  Yes |  |  |  | 🗸 | 🗸 | 🗸 |
| Cable | No  Yes |  |  |  | 🗸 | 🗸 | 🗸 |
| Residential internet | No  Yes |  |  |  |  |  | 🗸 |
| Direct TV/Netflix/Others | No  Yes |  |  |  |  |  | 🗸 |
| Bicycle | No  Yes | 🗸 | 🗸 | 🗸 | 🗸 | 🗸 | 🗸 |
| Motorcycle | No  Yes |  |  | 🗸 | 🗸 | 🗸 | 🗸 |
| Car | No  Yes |  |  | 🗸 | 🗸 | 🗸 | 🗸 |
| Sewing machine | No  Yes | 🗸 | 🗸 | 🗸 | 🗸 | 🗸 | 🗸 |
| Refrigerator | No  Yes | 🗸 | 🗸 | 🗸 | 🗸 | 🗸 | 🗸 |
| Washing machine | No  Yes |  |  |  |  | 🗸 | 🗸 |
| Microwave | No  Yes |  |  |  |  | 🗸 | 🗸 |
| Blender | No  Yes |  |  |  | 🗸 | 🗸 | 🗸 |
| Poultry | No  Yes |  |  | 🗸 | 🗸 | 🗸 | 🗸 |
| Pigs | No  Yes |  |  | 🗸 | 🗸 | 🗸 | 🗸 |

**Supplementary Note 2. Description of study waves and sample sizes at follow-up**

| **Study wave** | **Target sample** | **Households** | **Cohort members** | **Comment** |
| --- | --- | --- | --- | --- |
| *Village enumeration:* Village enumeration was carried out in the original four study villages. Information was collected on life status (alive, dead, unknown), location of residence and if residing in village, household assets. | | | | |
| 1967 | All households in village | 547 cohort households | 1634 | 567 households in total |
| 1975 | All households in village | 755 cohort households | 1942 | 755 households in total |
| 1987 | All households in village | 617 cohort households | 1360 | 1070 households in total |
| 1996 | All households in village | 650 cohort households | 853 | 1394 households in total |
| 2002 | All households in village | 820 cohort households | 1053 | 1671 households in total |
| *Study wave* | | | | |
| 1969-77 | Children < 7 years in study villages at start of trial and children born during the trial |  | 2392 |  |
| 1988-89 | Cohort members |  | 1577 | In or around study villages, Guatemala city |
| 1991-96 | All births in study villages |  | 458 | Study villages |
| 1996-99 | Children < 3y in study villages |  | 974 | Study villages |
| 1997-98 | Cohort members born during the trial and with at least 12 mo of infant data |  | 473 | In or around study villages, Guatemala city |
| 2002-04 | Cohort members |  | 1571 | Anywhere in Guatemala |
| 2005-07 | 3-generational family pedigrees |  | 1009 | In or around study villages, Guatemala city |
| 2015-16 | Cohort members | 1075 cohort households | 1163 | Anywhere in Guatemala |
| 2017-18 | Cohort members | 1145 cohort households | 1265 | Anywhere in Guatemala |

Additional information on cohort characteristics and follow-up have been published previously (1, 2).

**References for online-only supplementary material**

1. Stein AD, Melgar P, Hoddinott J, Martorell R. Cohort Profile: the Institute of Nutrition of Central America and Panama (INCAP) Nutrition Trial Cohort Study. Int J Epidemiol. 2008;37(4):716-20.

2. Ramirez-Zea M, Melgar P, Rivera JA. INCAP Oriente longitudinal study: 40 years of history and legacy. J Nutr. 2010;140(2):397-401.

**Supplementary Note 3: Comparison of approaches used to create asset indices**

|  | **Type of input variable** | **What is a factor/component?** | **What is the relation between components?** | **What does it explain?** | **What are variable loadings?** | **What is the estimation procedure?** | **What is the error variance?** |
| --- | --- | --- | --- | --- | --- | --- | --- |
| **Principal Component Analysis** | Continuous^1^ | Components are linear combinations of input variables and vice versa | Uncorrelated | Overall variance (diagonal and off-diagonal in correlation matrix) | Covariance of component and variable. | Eigen Decomposition or Singular Value Decomposition | Not applicable since this is included in variance explained by components |
| **Exploratory Factor Analysis** | Continuous^2^ | Underlying latent factor(s) which gives rise to input variables | Can be correlated or uncorrelated | Communalities (proportion of variance in input that is shared) | Regression coefficient (correlation) of input variable on factor | Different procedures^3^ (we use residual minimization) | Uniqueness (Considered as measurement error in latent variable model) |
| **Multiple Correspondence Analysis** | Dichotomous | Components explain inertia (how much values differ from expected if independent) | Uncorrelated | Overall inertia | Coordinates of centroids of categories of variables | Iterative; uses Singular Value Decomposition | Not applicable |

1 If data are multivariate normal, components are expected to be linearly independent (and uncorrelated by definition). If input variables are not multivariate-normally distributed, higher order dependencies between them could still exist.

2 Factor analysis assumes that the uniqueness (regression errors) for each input variable are normally distributed. It doesn’t assume each variable is normally distributed. However, extracting factors under maximum likelihood estimation assumes multivariate normality.

3 Principal axis factorization seeks to explain the common variance. Least squares estimation via residual minimization (iterative; unweighted or generalized/weighted procedures) seeks to explain the observed correlation matrix based on estimated matrix derived from factors. Maximum likelihood estimation assumes normally distributed factors which produce input variables that are correlated, with uniqueness (regression errors) being normally distributed.

**Supplementary Table 1. Definitions of durable assets and housing characteristics**

| **Item** | **Categorization for harmonized index** | **Categorization for S4** |
| --- | --- | --- |
| Radio | Yes, No | Yes, No |
| Record player | Yes, No | Yes, No |
| Sewing machine | Yes, No | Yes, No |
| Refrigerator | Yes, No | Yes, No |
| Television | Yes, No | Yes, No |
| Bicycle | Yes, No | Yes, No |
| Motorcycle | Yes, No | Yes, No |
| Automobile | Yes, No | Yes, No |
| Owns land | Yes, No | Yes, No |
| Owns house | Yes, No | Yes, No |
| Crowding (Rooms per member) | Continuous | Continuous |
| Floor quality | Low: Earth  High: Brick or clay, cement, mosaic, wood | Low: Earth  Medium: Brick or clay, cement  High: Mosaic, wood |
| Roof quality | Low: Thatched or similar  High: Tile, metal, concrete | Low: Thatched or similar  Medium: Tile, metal  High: Concrete |
| Wall quality | Low: Cane, mix of clay-cane-wood, mix of clay-cane-wood-cement  High: Mud brick (w/o cement), wooden, brick, metal | Low: Cane, mix of clay-cane-wood, mix of clay-cane-wood-cement, metal  Medium: Mud brick (w/o cement), wooden, metal  High: Brick |
| Separate kitchen | Low: No kitchen, in bedroom  High: Separate place, built-in | Low: No kitchen, in bedroom  Medium: Separate place  High: Built-in |
| Formal cooking medium | Low: No formal means, floor, low or high removable wood & charcoal stove  High: Low or high fixed wood & charcoal stove, wood/gas/electric stove | Low: Low: No formal means, floor  Medium: Low or high removable wood & charcoal stove, low or high fixed wood & charcoal stove  High: Wood/gas/electric stove |
| Sanitary installation | Yes: Latrine (pit/other), Toilet, Septic tank  No: No sanitary installation | Low: No sanitary installation  Medium: Latrine (pit/other)  High: Toilet, Septic tank |
| Electricity | Yes, No | Yes, No |
| Improved water source | High: Well or public water system  Low: Otherwise | High: Public water system  Medium: Well or public pile/pitchers  Low: Spring or river |
|  | **Additional assets for S5** |  |
| Video player | Yes, No |  |
| Sound system | Yes, No |  |
| Computer | Yes, No |  |
| Telephone | Yes, No |  |
| Washing machine | Yes, No |  |
| Sewage | High: Public system or septic tank  Low: Otherwise |  |

**Supplementary Table 2. Rank correlation with harmonized index on pairwise dropping of items**

|  | **Radio** | **Record Player** | **Sewing Machine** | **Refrigerator** | **Television** | **Bicycle** | **Motorcycle** | **Automobile** | **Owns land** | **Owns house** | **Rooms per member** | **High quality floor** | **High quality roof** | **High quality walls** | **Separate kitchen** | **Formal cooking medium** | **Sanitary installation** | **Electricity** | **Improved water source** |
| --- | --- | --- | --- | --- | --- | --- | --- | --- | --- | --- | --- | --- | --- | --- | --- | --- | --- | --- | --- |
| Radio | 0.99 |  |  |  |  |  |  |  |  |  |  |  |  |  |  |  |  |  |  |
| Record Player | 0.99 | 0.99 |  |  |  |  |  |  |  |  |  |  |  |  |  |  |  |  |  |
| Sewing Machine | 0.99 | 0.99 | 0.99 |  |  |  |  |  |  |  |  |  |  |  |  |  |  |  |  |
| Refrigerator | 0.97 | 0.97 | 0.97 | 0.97 |  |  |  |  |  |  |  |  |  |  |  |  |  |  |  |
| Television | 0.98 | 0.98 | 0.98 | 0.96 | 0.98 |  |  |  |  |  |  |  |  |  |  |  |  |  |  |
| Bicycle | 0.98 | 0.98 | 0.98 | 0.96 | 0.97 | 0.98 |  |  |  |  |  |  |  |  |  |  |  |  |  |
| Motorcycle | 0.98 | 0.98 | 0.98 | 0.97 | 0.97 | 0.97 | 0.98 |  |  |  |  |  |  |  |  |  |  |  |  |
| Automobile | 0.98 | 0.98 | 0.98 | 0.97 | 0.97 | 0.97 | 0.98 | 0.98 |  |  |  |  |  |  |  |  |  |  |  |
| Owns land | 0.99 | 0.99 | 0.98 | 0.97 | 0.98 | 0.98 | 0.98 | 0.98 | 0.99 |  |  |  |  |  |  |  |  |  |  |
| Owns house | 0.99 | 0.99 | 0.98 | 0.97 | 0.98 | 0.98 | 0.98 | 0.98 | 0.98 | 0.99 |  |  |  |  |  |  |  |  |  |
| Rooms per member | 0.99 | 0.99 | 0.99 | 0.97 | 0.99 | 0.99 | 0.99 | 0.98 | 0.99 | 0.99 | 0.99 |  |  |  |  |  |  |  |  |
| High quality floor | 0.98 | 0.98 | 0.98 | 0.96 | 0.97 | 0.97 | 0.98 | 0.98 | 0.98 | 0.98 | 0.98 | 0.98 |  |  |  |  |  |  |  |
| High quality roof | 0.99 | 0.99 | 0.98 | 0.97 | 0.98 | 0.98 | 0.98 | 0.98 | 0.98 | 0.99 | 0.99 | 0.98 | 0.99 |  |  |  |  |  |  |
| High quality walls | 0.98 | 0.98 | 0.98 | 0.97 | 0.97 | 0.98 | 0.98 | 0.98 | 0.98 | 0.98 | 0.99 | 0.98 | 0.98 | 0.98 |  |  |  |  |  |
| Separate kitchen | 0.99 | 0.99 | 0.98 | 0.97 | 0.98 | 0.98 | 0.98 | 0.98 | 0.98 | 0.98 | 0.99 | 0.98 | 0.98 | 0.98 | 0.99 |  |  |  |  |
| Formal cooking medium | 0.98 | 0.98 | 0.98 | 0.97 | 0.97 | 0.97 | 0.98 | 0.98 | 0.98 | 0.98 | 0.98 | 0.97 | 0.98 | 0.98 | 0.98 | 0.98 |  |  |  |
| Sanitary installation | 0.98 | 0.98 | 0.98 | 0.97 | 0.97 | 0.97 | 0.98 | 0.98 | 0.98 | 0.98 | 0.99 | 0.98 | 0.98 | 0.98 | 0.98 | 0.98 | 0.98 |  |  |
| Electricity | 0.98 | 0.98 | 0.98 | 0.97 | 0.97 | 0.97 | 0.98 | 0.98 | 0.98 | 0.98 | 0.99 | 0.98 | 0.98 | 0.98 | 0.98 | 0.98 | 0.98 | 0.98 |  |
| Improved water source | 0.98 | 0.98 | 0.98 | 0.97 | 0.97 | 0.97 | 0.98 | 0.98 | 0.98 | 0.98 | 0.99 | 0.98 | 0.98 | 0.98 | 0.98 | 0.98 | 0.98 | 0.98 | 0.98 |

**Supplementary Table 3. Rank correlation with harmonized index using 19 items on pairwise dropping of study waves**

|  | **1967** | **1975** | **1987** | **2002** | **2016** | **2018** |
| --- | --- | --- | --- | --- | --- | --- |
| 1967 | 0.97 |  |  |  |  |  |
| 1975 | 0.91 | 0.98 |  |  |  |  |
| 1987 | 0.96 | 0.96 | 0.98 |  |  |  |
| 2002 | 0.97 | 0.98 | 0.98 | 0.99 |  |  |
| 2016 | 0.98 | 0.99 | 0.99 | 0.99 | 0.99 |  |
| 2018 | 0.98 | 0.99 | 0.99 | 0.99 | 1.00 | 0.99 |

**Supplementary Table 4. Rank correlation with harmonized index using 19 items on joint dropping of study wave and asset**

|  | **1967** | **1975** | **1987** | **2002** | **2016** | **2018** |
| --- | --- | --- | --- | --- | --- | --- |
| Radio | 0.97 | 0.98 | 0.98 | 0.99 | 0.99 | 0.99 |
| Record Player | 0.97 | 0.98 | 0.98 | 0.99 | 0.99 | 0.99 |
| Sewing Machine | 0.97 | 0.98 | 0.98 | 0.98 | 0.99 | 0.99 |
| Refrigerator | 0.95 | 0.96 | 0.97 | 0.98 | 0.98 | 0.99 |
| Television | 0.95 | 0.96 | 0.97 | 0.98 | 0.99 | 0.99 |
| Bicycle | 0.96 | 0.96 | 0.97 | 0.98 | 0.99 | 0.99 |
| Motorcycle | 0.97 | 0.97 | 0.98 | 0.98 | 0.99 | 0.99 |
| Automobile | 0.96 | 0.97 | 0.98 | 0.98 | 0.99 | 0.99 |
| Owns land | 0.98 | 0.98 | 0.98 | 0.98 | 0.99 | 0.99 |
| Owns house | 0.97 | 0.98 | 0.98 | 0.99 | 0.99 | 0.99 |
| Rooms per member | 0.98 | 0.98 | 0.99 | 0.99 | 0.99 | 0.99 |
| High quality floor | 0.96 | 0.97 | 0.98 | 0.98 | 0.99 | 0.99 |
| High quality roof | 0.97 | 0.98 | 0.98 | 0.98 | 0.99 | 0.99 |
| High quality walls | 0.97 | 0.98 | 0.98 | 0.98 | 0.99 | 0.99 |
| Separate kitchen | 0.97 | 0.98 | 0.98 | 0.98 | 0.99 | 0.99 |
| Formal cooking medium | 0.97 | 0.97 | 0.98 | 0.98 | 0.99 | 0.99 |
| Sanitary installation | 0.97 | 0.97 | 0.98 | 0.98 | 0.99 | 0.99 |
| Electricity | 0.97 | 0.97 | 0.98 | 0.98 | 0.99 | 0.99 |
| Improved water source | 0.97 | 0.97 | 0.98 | 0.98 | 0.99 | 0.99 |

**Supplementary Figure 1. Distribution of harmonized asset index at study waves for all households included**


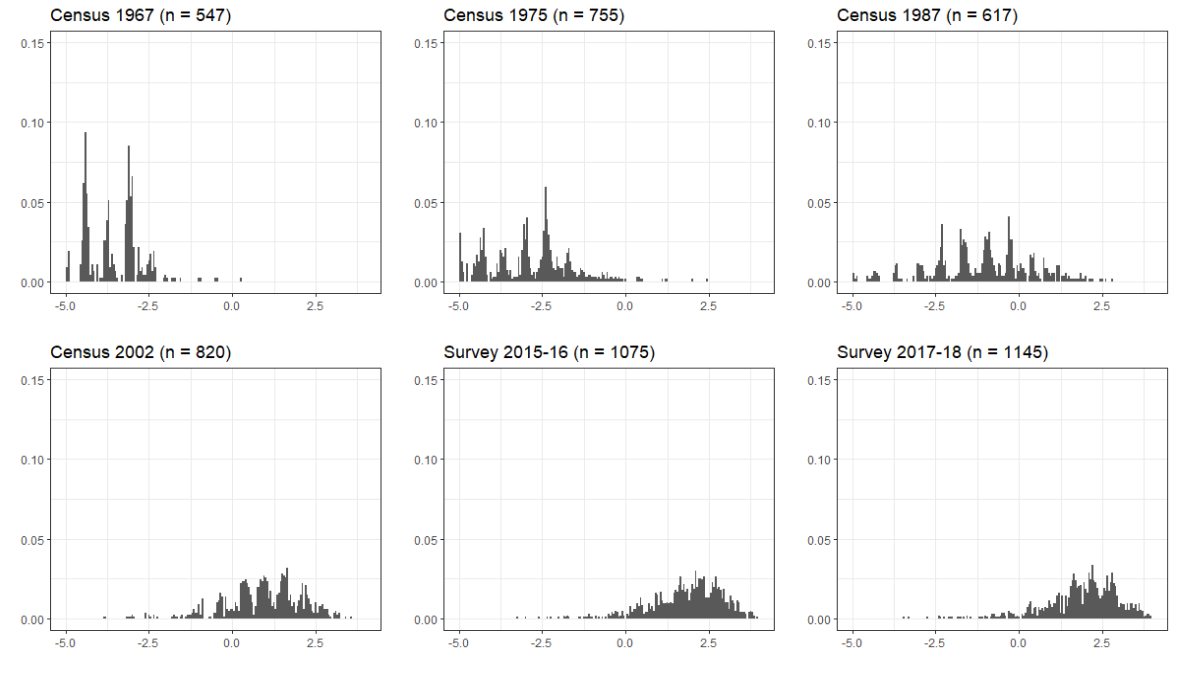


**Supplementary Figure 2. Flowchart of cohort members (n = 2392)**

**
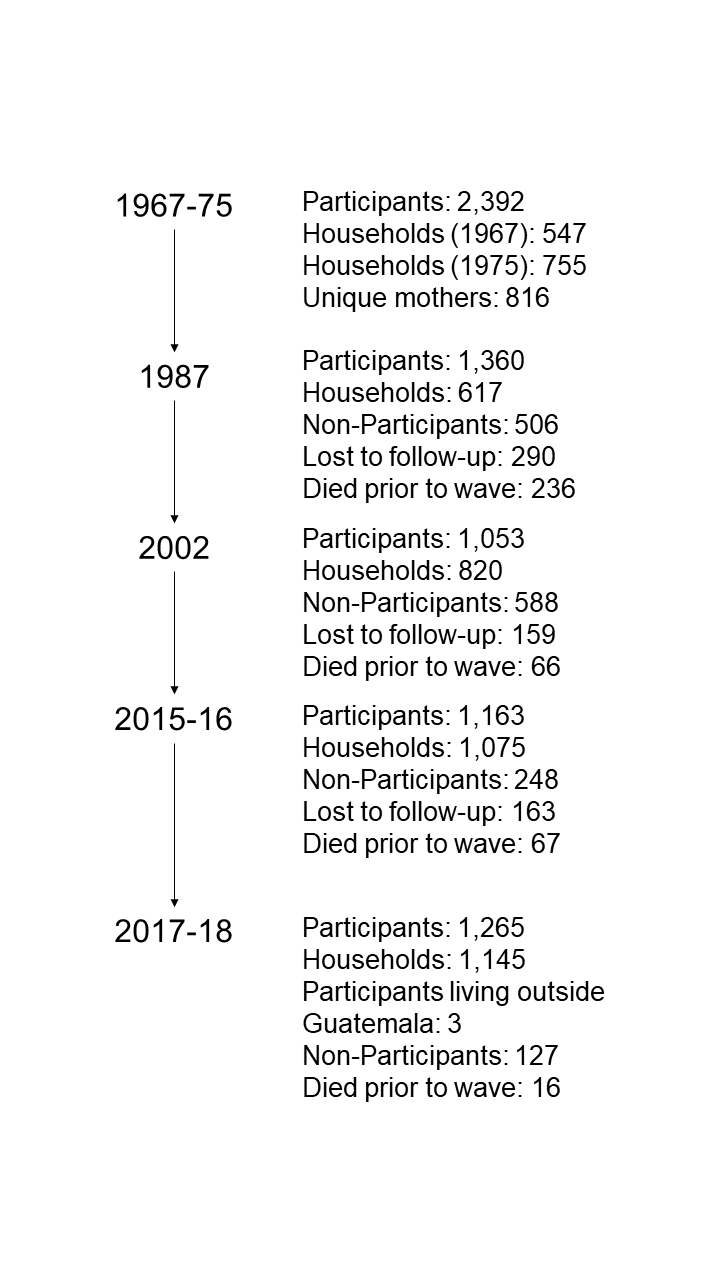
**

Participation in surveys of cohort members who were alive at the time and residing in Guatemala conducted in 1988-89 (Follow-up study) and 2002-04 (Human Capital study) were 1577 (73%) and 1571 (85%) respectively. The numbers of participants in the above flow-chart are for those for whom asset data in the census or study wave was available.
